# Anti-psychotic Nature of Antibiotics: Vancomycin and Omadacycline Combination Ameliorating Stress in a Zebrafish Model

**DOI:** 10.7759/cureus.56195

**Published:** 2024-03-14

**Authors:** Pavitra Shivani Mohan Raj, Taniya Mary Martin, Meenakshi Sundaram Kishore Kumar, Lavanya Prathap

**Affiliations:** 1 Department of Anatomy, Saveetha Medical College and Hospital, Saveetha Institute of Medical and Technical Sciences, Saveetha University, Chennai, IND; 2 Department of Anatomy, Saveetha Dental College and Hospital, Saveetha Institute of Medical and Technical Sciences, Saveetha University, Chennai, IND

**Keywords:** anti-psychotic drug, sustainable development, behavior analysis, zebrafish, omadacycline, oral vancomycin therapy

## Abstract

Background

Stress affects mental health significantly and is a ubiquitous feature of contemporary living. Among the possible antibiotics are omadacycline and vancomycin, whose anti-inflammatory properties have also been thoroughly documented in recent research. The goal of the current study was to examine their complex involvement in the brain’s stress response circuits and how they modulate stress. An established model organism that provides a useful platform for examining stress-induced behaviors and possible therapeutic approaches is the zebrafish. To investigate how dopamine affects the stress response, we used a zebrafish model that was exposed to stress.

Methodology

For three minutes, zebrafish were continually subjected to chasing stress. They were then given antibiotic combinations of 50 µg/mL each of vancomycin and omadacycline at various ratios of 1:1, 3:1, and 3:1. Behavior alterations, including freezing bouts, top-bottom ratios, and latency periods, were analyzed and contrasted with control groups. ImageJ software was utilized to analyze the video footage of the fish.

Results

The study showed that the combination of omadacycline and vancomycin greatly reduced the behaviors in zebrafish caused by stress. They chose their concentration (50 µg/mL) according to the lethal concentration 50% result. By shortening the latency time and increasing the intensity of breezing sessions, these chemicals restored almost normal activity. There was statistical significance in the outcomes. The results show that the combination of vancomycin and omadacycline may have an anti-psychotic impact on zebrafish behaviors brought on by stress. Their control of stress reactions is consistent with their known roles in the reward and stress circuits of the brain. These results emphasize the complex interactions between neurotransmitter systems and the control of stress, highlighting the therapeutic potential of dopamine in the treatment of stress-related mental illnesses.

Conclusions

The combination of vancomycin and omadacycline has been shown to have anti-psychotic effects, which presents potential opportunities for the development of new treatment strategies for mental diseases associated with stress. To fully understand the specific processes underpinning their involvement in stress management and how they relate to mental illnesses in humans, more investigation is necessary.

## Introduction

Modern human life now includes stress as a given. People frequently struggle with numerous sorts of stress, ranging from the pressures of job and personal duties to the fast-paced dynamics of modern life. Even though stress is an evolutionary adaptation to assist humans in overcoming obstacles, its prevalence in today’s society has raised questions about how it may affect people’s health [1]. To negotiate the complexity of contemporary life and preserve mental and physical health, it is essential to understand the origins, effects, and coping methods associated with stress. Nature frequently experiences stress, which affects a variety of organisms, including aquatic species such as zebrafish (*Danio rerio*). Zebrafish have become an important model organism in biomedical research because of their genetic similarity to humans, rapid development, and optical transparency of embryos, which permits real-time imaging of physiological processes. Zebrafish are a good model for researching stress-related phenomena and potential treatment methods because they have a well-preserved stress response system that is similar to that of higher vertebrates [2].

Stress can have a detrimental impact on various aspects of our lives, affecting both our physical and mental well-being. Physically, it can lead to conditions such as headaches, digestive problems, and disruptions in sleep patterns. On a psychological and emotional level, stress can manifest as confusion, anxiety, and depression. When chronic stress goes untreated, which refers to persistent and long-lasting stress, it can further contribute to serious health issues such as high blood pressure and a compromised immune system [3]. These health consequences underscore the importance of managing and addressing stress to maintain overall well-being. In aquatic habitats, zebrafish face a variety of stressors such as temperature variations, water quality, social interactions, and disease exposure. These stressors might cause a “stress response” defined by the production of stress hormones such as cortisol, which aids the fish in coping with the harsh environment [2,3]. Although the stress response is an essential survival mechanism, prolonged or excessive stress can have negative consequences on zebrafish immunity, behavior, physiology, and general health. Crowded circumstances, handling, transportation, and other stressors in aquaculture can result in fish health problems and slow growth rates which can have a significant impact on the productivity of aquaculture systems as a whole [4]. As a result, there is an increasing interest in developing new methods of reducing stress in fish raised in aquaculture. An effective remedy for bacterial infections, particularly those caused by Gram-positive bacteria, is the glycopeptide antibiotic vancomycin. It works by preventing the formation of bacterial cell walls, making it an antibiotic that is efficient against a variety of infections [5]. Vancomycin, a drug well-known for its ability to combat germs, may potentially offer other advantages, such as lowering inflammation and serving as an antioxidant, according to recent research. A more recent antibiotic that functions against some illnesses caused by Gram-positive bacteria and is comparable to vancomycin, omadacycline, has been licensed for use in human medicine. Similar to vancomycin, omadacycline has been shown in studies to decrease inflammation, which suggests that it may be a useful treatment for resistant infections. Its efficacy in treating a range of illnesses may be attributed to its capacity to combat inflammation [6]. It would be interesting to investigate whether vancomycin and omadacycline could extend their protective benefits to reduce stress responses in zebrafish given their possible anti-inflammatory qualities. Therefore, the goal of this work is to examine the anti-stress activity of vancomycin and omadacycline in a zebrafish model to provide insights into their prospective uses as stress-relieving substances in aquaculture settings [7]. This study holds significant implications for the aquaculture industry, as the development of effective anti-stress therapies can enhance fish health and welfare, leading to improved productivity and sustainability. Moreover, investigating the pharmacological properties of antibiotics beyond their antimicrobial effects could uncover new therapeutic opportunities and contribute to broader advancements in stress management and aquaculture practices.

## Materials and methods

Zebrafish were housed in a controlled environment with a 12-hour light/12-hour dark cycle at 25°C and were fed twice daily. The fish used in the study were aged for approximately eight months. The lethal concentration 50% values were determined for vancomycin and omadacycline following the OECD guidelines for fish toxicity testing (No. 203) [8]. The procedure was approved by the Institutional Animal Ethical Committee, Saveetha Dental College and Hospitals, Saveetha Institute of Medical and Technical Sciences (approval number: BRULAC/SDCH/SIMATS/IAEC/06-2023/15).

The distinct stress-inducing method, the chasing method (for three minutes) was employed for stressing the fish. Each fish underwent a designated stress-inducing procedure and was promptly transferred to the pretreatment beaker for 20 minutes. Subsequently, the fish was introduced into the unique tank setup, and their behavior was assessed. The evaluation involved recording and analyzing distinct behavioral endpoints, as defined and interpreted by previous methods [9]. To assess the stress response in zebrafish, a transparent rectangular tank (28 cm length × 7 cm width × 15 cm depth) was employed. The tank, holding approximately 3 L of water, was equipped with cameras for the fish’s movement. The setup was positioned on a stable surface [10]. For pretreatment, a 1,000 mL translucent beaker was utilized. Alprazolam was used as a positive control (5 and 10 µg/mL, respectively). The behavioral endpoints observed and their respective implications in the context of anxiety levels in zebrafish (n = 12) are summarized in Table 1. These experimental designs were influenced by previous stress response modeling studies in zebrafish [11].

**Table 1:** Behavioral endpoints and the effects on zebrafish anxiety levels.

Endpoint (units)	Definition	Interpretation
Latency to enter the top (seconds)	Time taken for the first center-of-mass crossing from the defined bottom to the top of the novel tank	Longer latency signifies higher anxiety levels as zebrafish tend to explore gradually from the tank’s bottom in novel environments
Time spent in the top (seconds)	Total duration spent in the upper portion of the novel tank	An extended duration at the tank’s top indicates lower anxiety levels as zebrafish exhibit greater comfort in this area
Time spent top:bottom ratio	The ratio of time spent on the top to time spent at the bottom	A lower ratio signifies elevated anxiety levels
Number of entries to the top	Count of crossings from the defined bottom portion to the top of the novel tank	More frequent top entries indicate reduced anxiety levels
Number of entries to the bottom	Count of crossings from the defined top portion to the bottom of the novel tank	Higher bottom entries suggest heightened anxiety levels
Entries top:bottom ratio	The ratio of the number of entries at the top to the number of entries at the bottom	A lower ratio points toward increased anxiety
Average entry duration (seconds)	Time spent on average at the top during each crossing	A shorter average entry duration suggests higher anxiety levels
Number of erratic movements	Instances of abrupt direction changes or repeated darting	Reflects heightened fear/anxiety, typically more prominent in stressed zebrafish
Freezing bouts (frequency)	Instances of complete immobility (>1 second), excluding the eyes and gills	Indicates increased anxiety and is more frequent in stressed zebrafish
Freezing duration (seconds)	Total duration of all freezing bouts	Reflects heightened anxiety and is generally longer in stressed zebrafish

## Results

**Figure 1:**
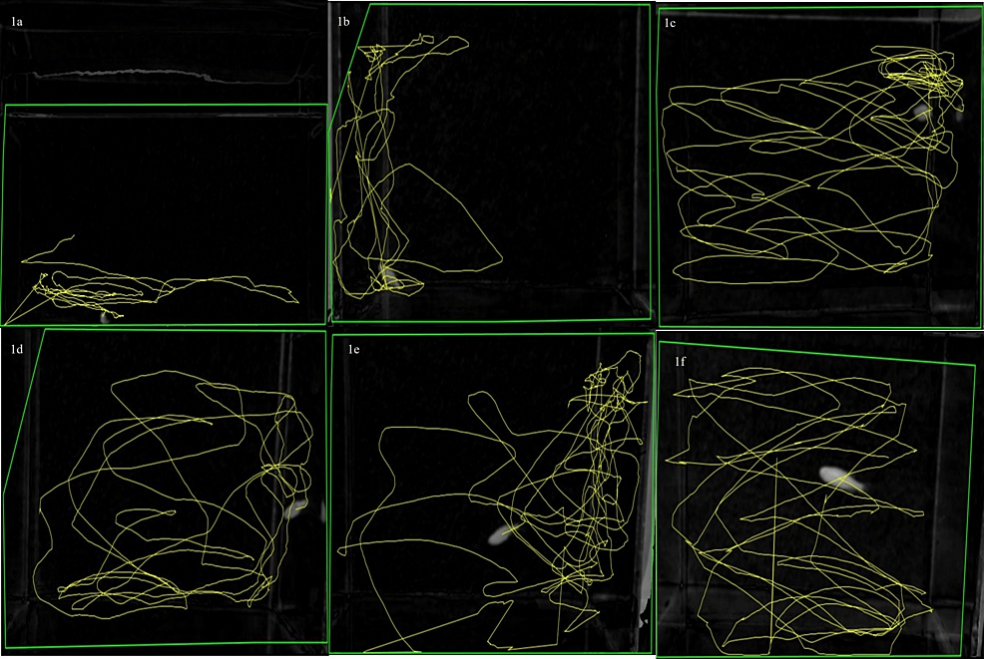
Comparison of control and treated fishes in their stress-induced condition. Stress was induced using the chasing method. The control group (a) revealed stress behavior. Alprazolam, used as a positive control, at 5 µg/mL and 10 µg/mL (b and c, respectively) showed better anti-anxiolytic activity compared to the control group.  The omadacycline and vancomycin combination showed comparable activity as alprazolam (d, e, and fL 1:1, 1:3, and 3:1 combinations, respectively).

Fish in the control group (Figure 1a) displayed a stressed (chasing-induced) behavior witnessed by a longer duration for entering from the bottom to the top (latency period). This indicated that the fish felt more anxiety about exploring newer environments. Additionally, it was noted that the fish’s latency duration was greater than a minute and alprazolam altered its stressed behavior (Figure 1b). Alprazolam, at 5 µg/mL, made more displacement on fish movement than the control (Figure 1b). Similarly, at 10 µg/mL (Figure 1c), it had higher anxiolytic activity than control and 5 µg/mL. These results showed that alprazolam acted in a dose-dependent manner. Likewise, more displacement showed lesser stress behavior which was also ensured in the stress-induced antibiotic combination-treated group. The displacement increased with the increased concentration of the antibiotic combinations (Figures 1d, 1e). Further, they were characterized by fewer freezing bouts and periods of total immobility compared to controls that had elevated anxiety. The tracking analysis also revealed that antibiotic combinations of vancomycin and omadacycline (50 µg/mL each) at different ratios of 1:1, 3:1, and 3:1 produced better anxiolytic activity than controls compared to alprazolam (Figures 1a-1f). The results showed dose dependency. The number of erratic movements (such as the sudden direction changes and repetitive darting) was higher in controls than in the alprazolam and antibiotic combination-treated groups (Figures 1a-1f), respectively.

**Figure 2:**
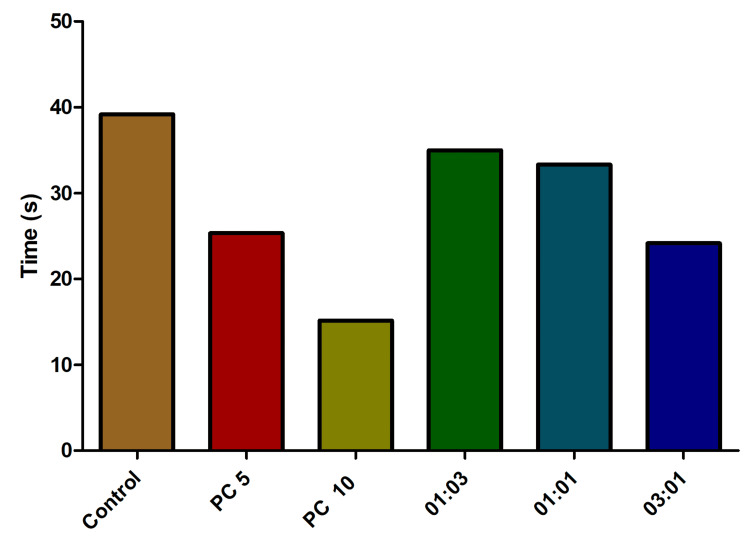
Impact of latency behavior (entries to the bottom) in fish treated with various combinations of omadacycline and vancomycin. Stress was induced using the chasing method. Alprazolam (PC) was used as a positive control at 5 µg/mL and 10 µg/mL.  Different combinations (1:3, 1:1, and 3:1) of omadacycline and vancomycin were used (50 µg/mL each) (data represented as mean ± standard error (SE), one-sample t-test, α = 0.05, p = 0.0001, significant). Image credit: Lavanya Prathap.

In the chasing stress experiment, the control group of fish displayed the lengthiest latency (Figure 2). The application of alprazolam as a positive control showed a 15.2% and 8.5% decrease (approximately) in latency time at 5 µg/mL and 10 µg/mL, respectively. The fish treated with the antibiotic combinations (1:1, 3:1, and 1:3) displayed a regular decreased latency pattern (dose-dependent) during the chasing experiment and were found to be statistically significant (one-sample t-test, p = 0.0001).

**Figure 3:**
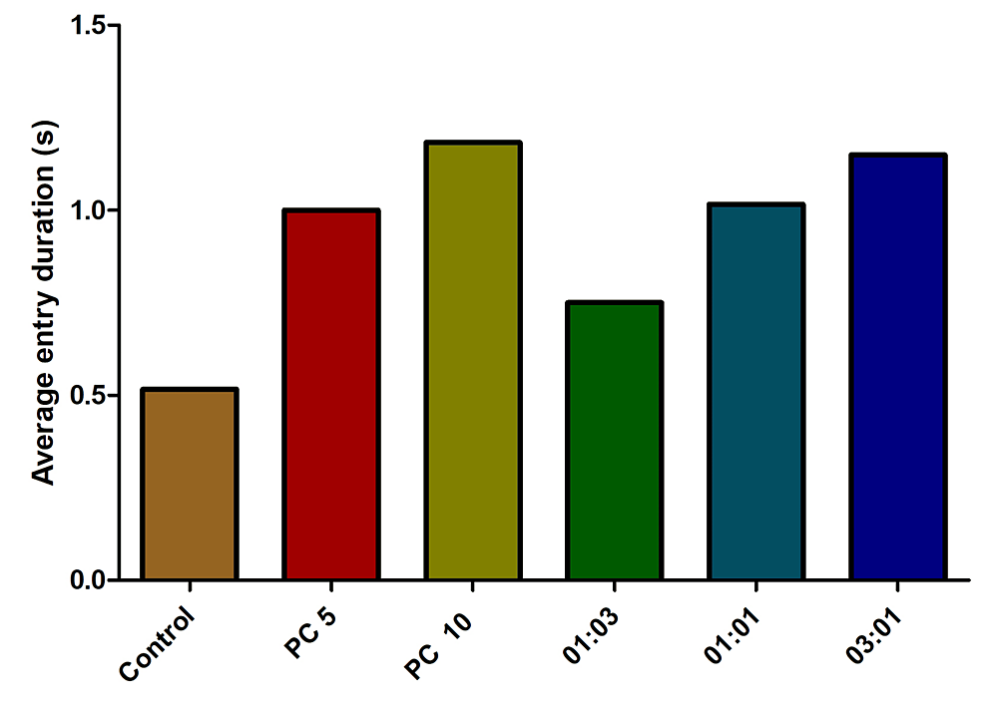
Time spent on the top behavior in fish treated with different combinations of omadacycline and vancomycin. Stress induction was performed using the chasing (C) method. Alprazolam (PC) was employed as a positive control at concentrations of 5 µg/mL and 10 µg/mL. Different combinations of omadacycline and vancomycin (at ratios of 1:3, 1:1, and 3:1) were administered at a concentration of 50 µg/mL each (data represented as mean ± standard error (SE), one-sample t-test, α = 0.05, p = 0.0003, significant). Image credit: Lavanya Prathap.

**Figure 4:**
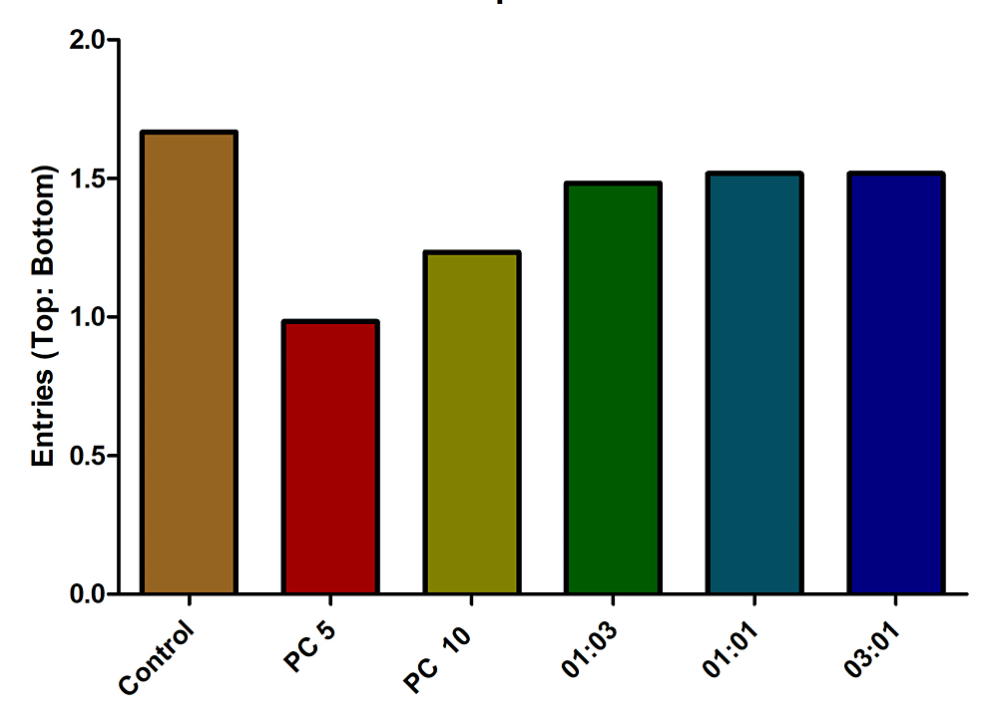
Impact of the top-to-bottom ratio behavior in fish treated with various combinations of omadacycline and vancomycin. Stress induction was achieved using the chasing (C) method. Alprazolam (PC) served as a positive control with concentrations of 5 µg/mL and 10 µg/mL. Different combinations of omadacycline and vancomycin (ratios 1:3, 1:1, and 3:1) were administered at a concentration of 50 µg/mL each. (data represented as mean ± standard error (SE), one-sample t-test, α = 0.05, p = 0.0003, significant). Image credit: Lavanya Prathap.

Regarding the duration spent in the upper section of the tank, a discernible dose-dependent effect was not observed either with alprazolam or antibiotic combinations-treated fish. Nevertheless, across the experiments, both alprazolam and antibiotic combinations-treated fish exhibited an elevated duration spent in the upper portion of the tank, as evidenced by higher values (Figures 3, 4).

As anticipated, based on the data collected during the observation period, it was evident that the fish treated with alprazolam and antibiotic combinations exhibited a notably higher top-to-bottom ratio compared to the control group, particularly at the highest concentration. Interestingly, the enhanced ratio in fishes treated with the 3:1 and 1:1 combinations of antibiotics further underscores the potential synergistic effects of these compounds. The observed elevation in the ratio, particularly under conditions of a concentration-dependent response, with the highest concentration (50 µg/mL) resulted in the most pronounced effects as previously reported (Figure 4).

**Figure 5:**
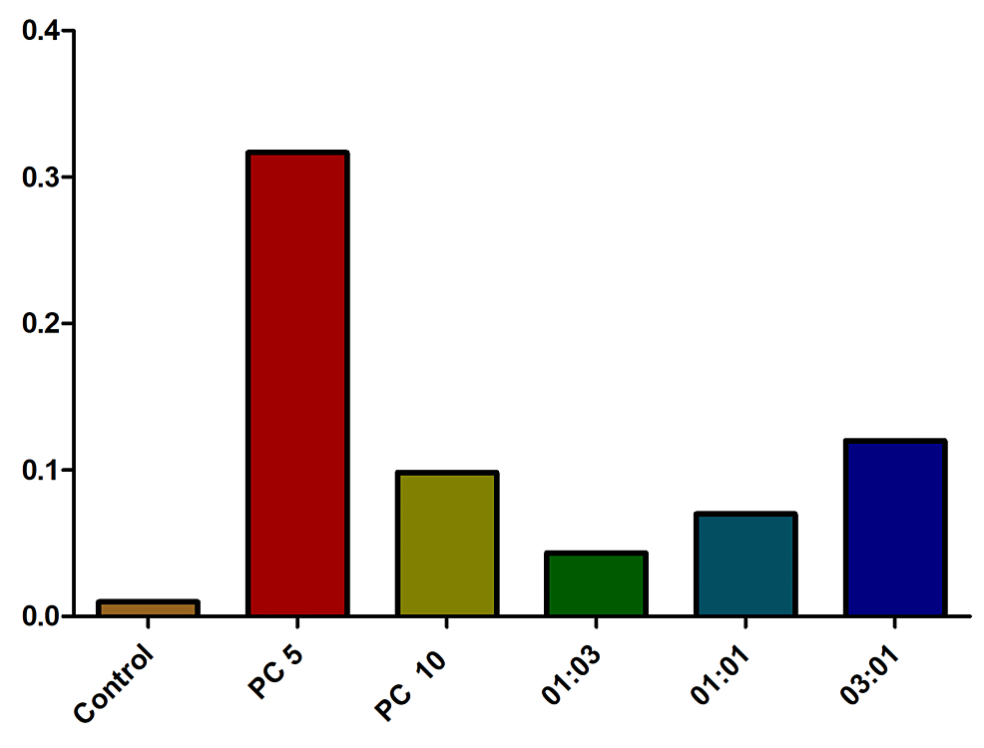
Influence of the number of entries to the top behavior in fish exposed to various combinations of omadacycline and vancomycin. Stress induction was achieved through the chasing (C) method. Alprazolam (PC) was utilized as a positive control, applied at concentrations of 5 µg/mL and 10 µg/mL. Diverse combinations of omadacycline and vancomycin (in ratios of 1:3, 1:1, and 3:1) were administered at a concentration of 50 µg/mL each (data represented as mean ± standard error (SE), one-sample t-test, α = 0.05, p = 0.056, not significant). Image credit: Lavanya Prathap.

In the context of chasing, the increased frequency of ascents could be attributed to the anxiolytic properties of alprazolam and the potentially stress-reducing effects of antibiotic combinations. These substances might modulate the fish’s stress responses, leading to heightened exploration of the water’s surface as a coping strategy. The absence of a dose-dependent effect in the chasing experiment suggests that the fish’s response to external stimuli, such as the presence of a potential threat, may not be influenced by the same mechanisms as their surface-oriented behaviors (Figure 5).

**Figure 6:**
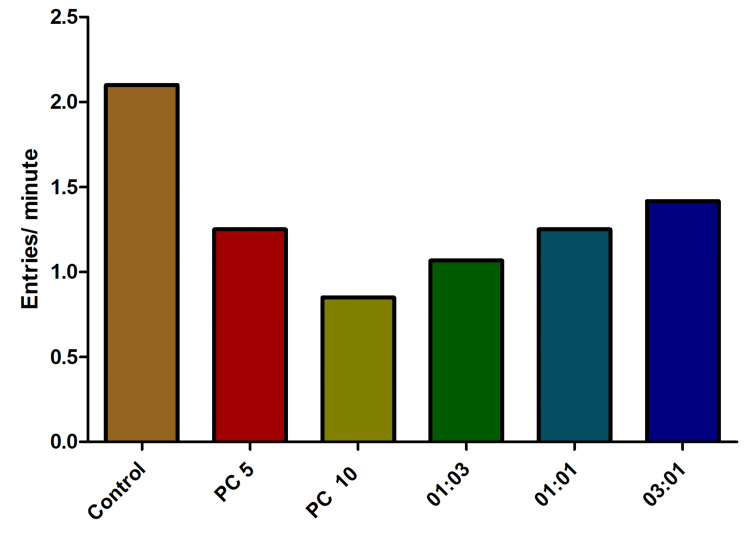
Impact of entries toward the bottom behavior in fish exposed to different combinations of omadacycline and vancomycin. Stress was induced using the chasing (C) method. Alprazolam (PC) was used as a positive control, applied at concentrations of 5 µg/mL and 10 µg/mL. Various combinations of omadacycline and vancomycin (ratios 1:3, 1:1, and 3:1) were administered at a concentration of 50 µg/mL each (data represented as mean ± standard error (SE), one-sample t-test, α = 0.05, p = 0.0006, significant). Image credit: Lavanya Prathap.

In all of our experiments, we observed results that achieved statistical significance. Fish treated with both alprazolam and antibiotic combinations exhibited a consistent reduction in the frequency of entries toward the bottom, and this trend followed a dose-dependent pattern (Figure 6).

**Figure 7:**
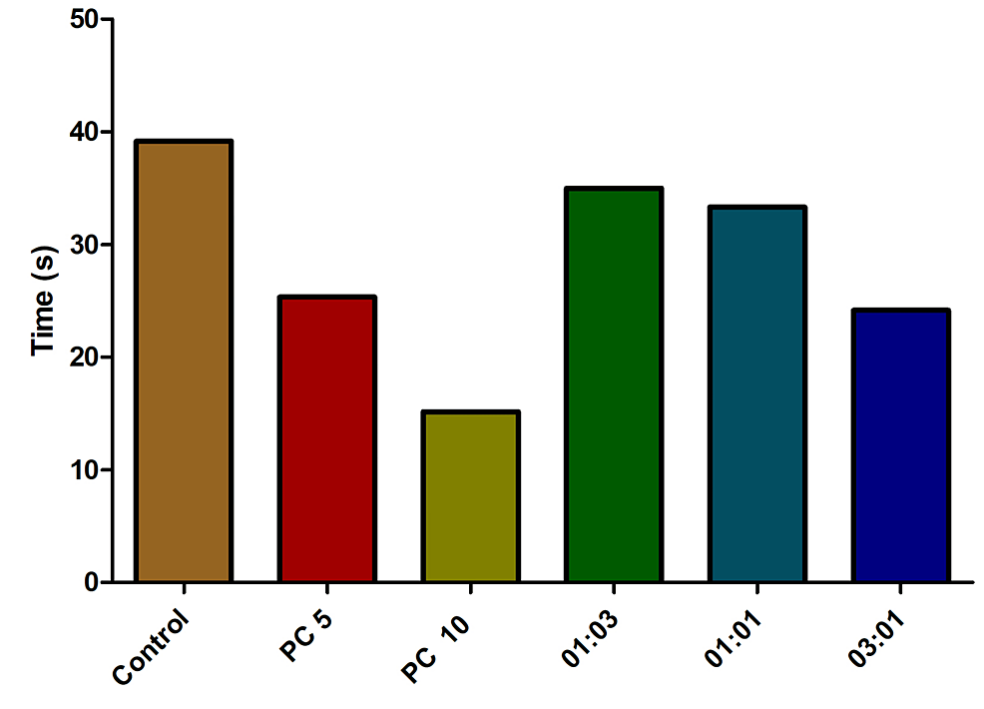
Impact of time spent on the bottom in fish exposed to varied omadacycline and vancomycin combinations. Stress was induced using the chasing method. Alprazolam (PC) was used as a positive control at concentrations of 5 µg/mL and 10 µg/mL. A range of omadacycline and vancomycin combinations (with ratios of 1:3, 1:1, and 3:1) was introduced at a consistent concentration of 50 µg/mL each (data represented as mean ± standard error (SE), one-sample t-test, α = 0.05, p = 0.001, significant). Image credit: Lavanya Prathap.

Consistent with the findings from the analyses of both the number of entries to the top and the number of entries to the bottom, the calculation of the top-to-bottom ratio yielded statistically significant outcomes. This observed trend of significance was maintained across all three experiments, displaying a consistent dose-dependent pattern (Figure 7).

**Figure 8:**
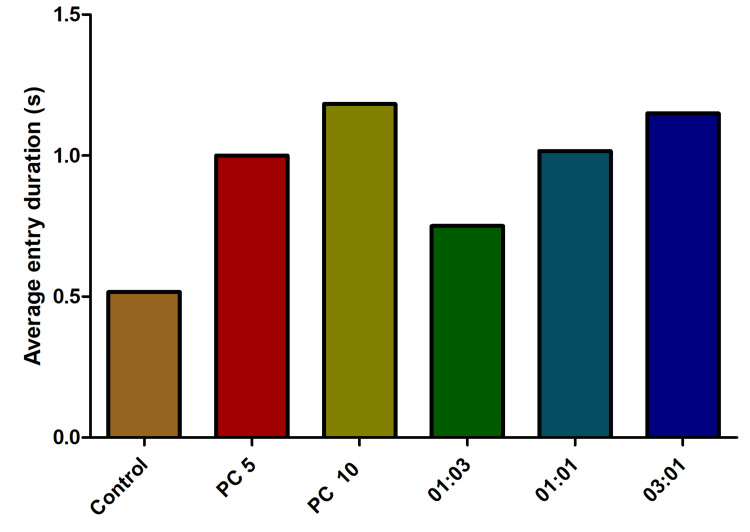
The effect of average entry duration behavior in fish exposed to different combinations of omadacycline and vancomycin. The fish were subjected to stress using the chasing method. As a positive control, alprazolam (PC) was applied at concentrations of 5 µg/mL and 10 µg/mL. A range of combinations of omadacycline and vancomycin (with ratios of 1:3, 1:1, and 3:1) were administered at a concentration of 50 µg/mL each (data represented as mean ± standard error (SE), one-sample t-test, α = 0.05, p = 0.0003, significant). Image credit: Lavanya Prathap.

The analysis of the average entry duration endpoint did not yield statistically significant findings. Contrary to the anticipated outcomes, the average entry duration was observed to be lower in the control group compared to the alprazolam-treated group. Notably, antibiotic combinations exhibited an increased average entry duration (Figure 8).

**Figure 9:**
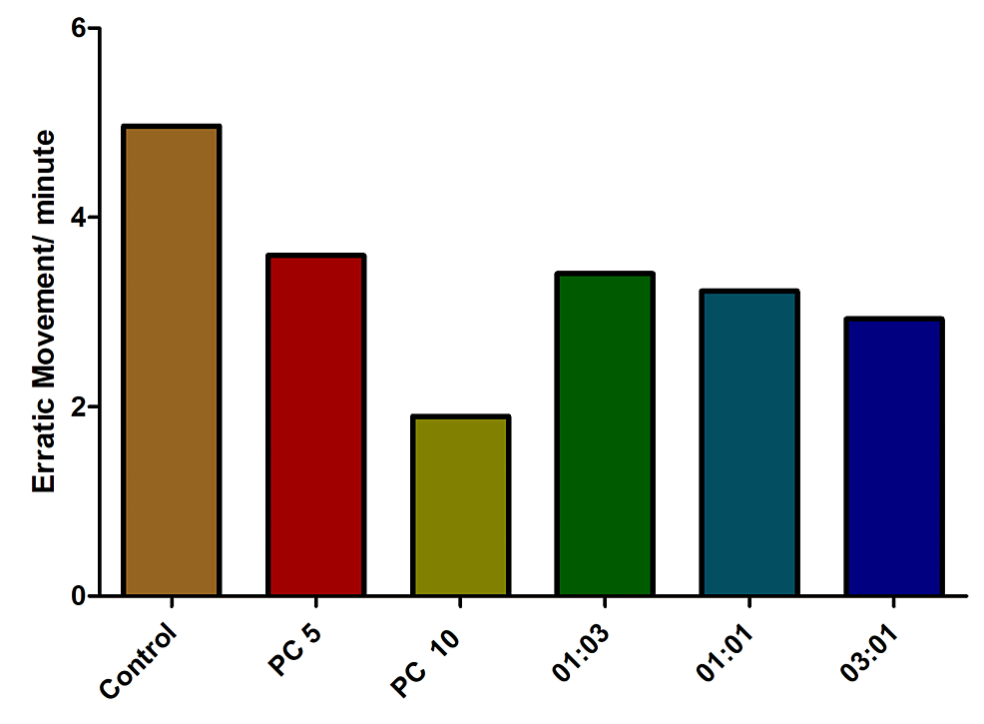
The influence of the number of erratic movements in fish exposed to varied omadacycline and vancomycin combinations. The experimental setup involved subjecting the fish to stress through the chasing method. Alprazolam (PC) was utilized as a positive control, administered at concentrations of 5 µg/mL and 10 µg/mL. A spectrum of omadacycline and vancomycin combinations (with ratios of 1:3, 1:1, and 3:1) were introduced at a uniform concentration of 50 µg/mL each (represented as mean ± standard error (SE), one-sample t-test, α = 0.05, p = 0.0004, significant). Image credit: Lavanya Prathap.

A noticeable reduction in the frequency of erratic movements was observed among fishes treated with both alprazolam and antibiotic combinations. Nonetheless, a discernible dose-dependent trend in this reduction was evident exclusively in the context of chase experiments (Figure 9).

**Figure 10:**
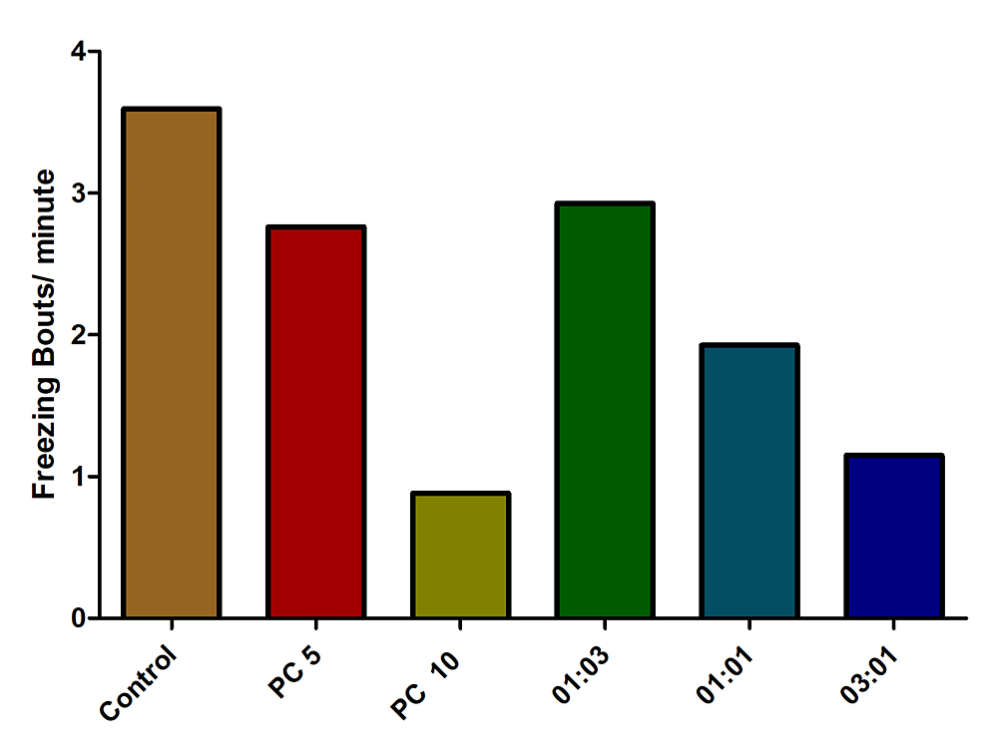
The impact of freezing bouts in fish exposed to varied combinations of omadacycline and vancomycin. The experimental design encompassed the imposition of stress on the fish using the chasing method. Alprazolam (PC) was employed as a positive control, administered at concentrations of 5 µg/mL and 10 µg/mL. A range of omadacycline and vancomycin combinations (with ratios of 1:3, 1:1, and 3:1) was introduced at a consistent concentration of 50 µg/mL each (data represented as mean ± standard error (SE), one-sample t-test, α = 0.05, p = 0.0039, significant). Image credit: Lavanya Prathap.

**Figure 11:**
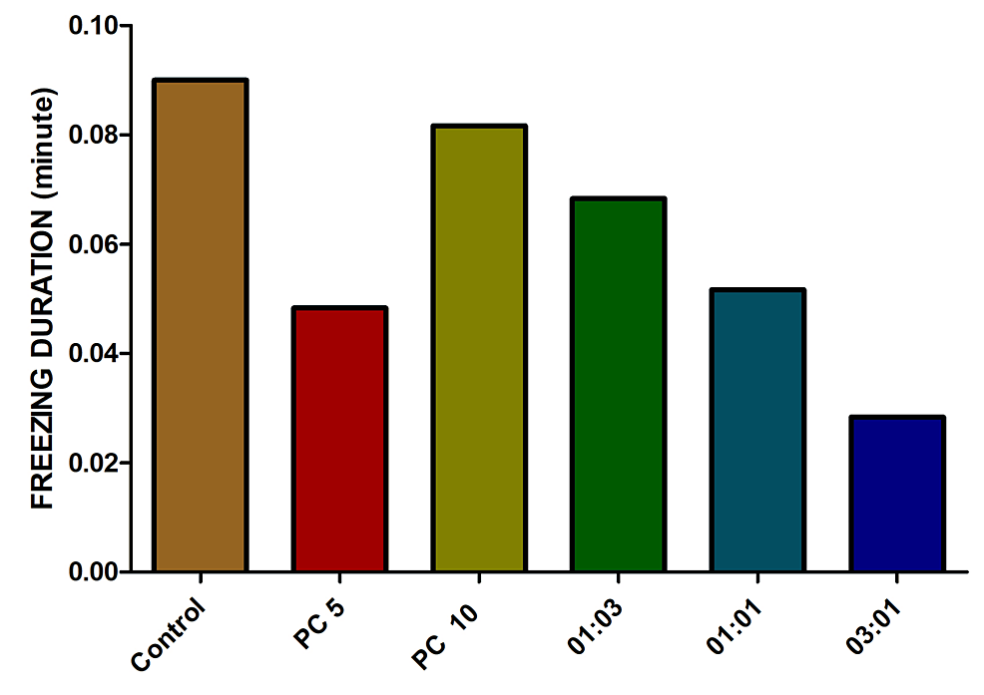
The impact of freezing duration in fish exposed to varied combinations of omadacycline and vancomycin. The experimental design encompassed the imposition of stress on the fish using the chasing method. Alprazolam (PC) was employed as a positive control, administered at concentrations of 5 µg/mL and 10 µg/mL. A range of omadacycline and vancomycin combinations (with ratios of 1:3, 1:1, and 3:1) was introduced at a consistent concentration of 50 µg/mL each (data represented as mean ± standard error (SE), one-sample t-test, α = 0.05, p = 0.001, significant). Image credit: Lavanya Prathap.

Both of these observed endpoints exhibited results that diverged from the initially anticipated values. Surprisingly, freezing bouts and their corresponding durations were notably elevated in the groups of fishes treated with alprazolam and antibiotic combination in comparison to the control group (Figures 10, 11).

## Discussion

During stress application, the fish displayed conspicuous signs of stress-related behavior, notably, an extended latency period when transitioning from the bottom to the top of the novel tank [12]. This prolonged latency suggests heightened anxiety levels in the fish, as zebrafish typically exhibit greater exploration in the tank’s bottom when introduced to unfamiliar environments. Calculating the ratio of time spent at the top versus the bottom of the tank, we observed a notably lower ratio, further indicating elevated anxiety levels. Fish with increased anxiety levels tend to avoid the top section and prefer staying at the bottom [13]. Conversely, fish displaying reduced anxiety exhibited more frequent entries into the top area, while those with heightened anxiety showed a predilection for frequent entries to the bottom. Additionally, we noted a conspicuous number of erratic movements, characterized by sudden changes in direction or repeated darting. These behaviors are indicative of heightened anxiety levels and are more prominently exhibited by stressed fish. Furthermore, the images revealed the presence of freezing bouts at various locations within the tank. These freezing bouts, characterized by complete immobility, excluding eye and gill movement, are a clear sign of increased anxiety [14]. Notably, stressed fish exhibited a higher frequency of freezing bouts, and the total duration of these immobile episodes was generally longer compared to non-stressed zebrafish [15]. In summary, the observed behaviors, i.e., latency to enter specific tank areas, top-to-bottom ratio, erratic movements, and freezing bouts, all provided consistent indicators of anxiety levels, with stressed fish consistently displaying more pronounced signs of anxiety than their non-stressed counterparts. Investigating the possible anti-stress properties of antibiotics such as vancomycin and omadacycline in zebrafish models provides fascinating new information about how stress is managed. Although antibiotics are typically used to treat bacterial infections, recent research suggests that they may also play a role in regulating stress responses by interacting with the gut microbiota. Due to the wider consequences of antibiotic usage, it is crucial to view these findings with caution and ethical considerations. Zebrafish are a useful model for studying stress because of their physiological similarity to humans [12]. Similar to how humans react to stimuli, their bodies and behaviors alter as a result. Because of this resemblance, researchers can examine how antibiotics can impact this model’s stress reactions [13-15].

Recent research indicates that the gut-brain axis plays a role in how stress is regulated by gut microbiota. Stress-related diseases have been linked to changes in gut microbial composition. Because of its potential to affect these microbiota-driven pathways and modify stress responses, vancomycin may have anti-stress properties [16]. The gut microbiota has been shown to interact with omadacycline, a tetracycline-class antibiotic effective against a variety of bacteria. This has motivated researchers to examine its possible impact on stress. We may discover novel pathways for omadacycline’s anti-stress effects by examining how it alters the composition of the gut microbiota and influences stress responses. The exploration of antibiotics such as vancomycin and omadacycline as potential regulators of stress responses in zebrafish models unveils a captivating dimension in stress management research. Traditionally known for their role in combating bacterial infections, antibiotics are now under scrutiny for their potential involvement in stress modulation through interactions with the gut microbiota. However, the broader implications of antibiotic use demand a balanced perspective and ethical consideration [17]. Shifts in gut microbial composition have been associated with stress-related diseases. Given vancomycin’s potential to influence these microbiota-driven pathways and subsequently modify stress responses, it is plausible that the antibiotic possesses anti-stress properties [18]. Furthermore, the delicate balance of the gut microbiota, which is increasingly associated with mood and mental health, can be upset by antibiotics. Anxiety and other mental diseases have been linked to changes in the makeup of the gut microbiota [19].

While the notion of antibiotics as potential regulators of stress holds promise, it is essential to approach these findings with caution. Meanwhile, the present study only included a limited sample (n = 12). This should be further analyzed with a larger sample. The widespread use of antibiotics has implications for microbial ecosystems, including the potential for antibiotic resistance and disruptions in microbial balance. Moreover, the ethical dimensions of utilizing antibiotics for stress modulation warrant thoughtful consideration.

The chasing experiment likely involves a more immediate and reflexive response that might not be as modulated by the concentrations of alprazolam and antibiotic combinations. It is important to consider that while these findings provide valuable insights, additional research is warranted to elucidate the underlying physiological, neurological, and behavioral mechanisms driving the observed effects. Furthermore, understanding the ecological implications of altered surface-oriented behaviors is crucial, as it can impact interactions with predators, prey, and other ecological variables.

## Conclusions

Vancomycin and omadacycline combination significantly reduced the stress level in zebrafish models. The present study allows researchers to gain valuable insights into the stress-regulating activity of antibiotic combinations This investigation not only sheds light on the intricate and interconnected web of stress regulation but also prompts several key considerations for further study and ethical deliberation.
